# A core outcome set for adult cardiac surgery trials: A consensus study

**DOI:** 10.1371/journal.pone.0186772

**Published:** 2017-11-02

**Authors:** Carina Benstoem, Ajay Moza, Patrick Meybohm, Christian Stoppe, Rüdiger Autschbach, Declan Devane, Andreas Goetzenich

**Affiliations:** 1 Department of Thoracic and Cardiovascular Surgery, University Hospital RWTH Aachen, Aachen, Germany; 2 Department of Anaesthesiology, Intensive Care and Pain Medicine, University Hospital Frankfurt, Frankfurt am Main, Germany; 3 Department of Intensive Care Medicine, University Hospital RWTH Aachen, Aachen, Germany; 4 Department of Nursing and Midwifery, National University of Ireland Galway, School of Nursing and Midwifery, Galway, Ireland; Harvard Medical School, UNITED STATES

## Abstract

**Background:**

Invasive off- or on-pump cardiac surgery (elective and emergency procedures, excluding transplants are routinely performed to treat complications of ischaemic heart disease. Randomised controlled trials (RCT) evaluate the effectiveness of treatments in the setting of cardiac surgery. However, the impact of RCTs is weakened by heterogeneity in outcome measuring and reporting, which hinders comparison across trials. Core outcome sets (COS, a set of outcomes that should be measured and reported, as a *minimum*, in clinical trials for a specific clinical field) help reduce this problem. In light of the above, we developed a COS for cardiac surgery effectiveness trials.

**Methods:**

Potential core outcomes were identified a priori by analysing data on 371 RCTs of 58,253 patients. We reached consensus on core outcomes in an international three-round eDelphi exercise. Outcomes for which at least 60% of the participants chose the response option “no” and less than 20% chose the response option “yes” were excluded.

**Results:**

Eighty-six participants from 23 different countries involving adult cardiac patients, cardiac surgeons, anaesthesiologists, nursing staff and researchers contributed to this eDelphi. The panel reached consensus on four core outcomes: 1) Measure of mortality, 2) Measure of quality of life, 3) Measure of hospitalisation and 4) Measure of cerebrovascular complication to be included in adult cardiac surgery trials.

**Conclusion:**

This study used robust research methodology to develop a minimum core outcome set for clinical trials evaluating the effectiveness of treatments in the setting of cardiac surgery. As a next step, appropriate outcome measurement instruments have to be selected.

## Introduction

Ischaemic heart disease is the number one cause of death worldwide and thus a major contributor to the global the burden of disease [1]. Cardiac surgery is routinely performed to treat complications of this disease and RCTs evaluate the effectiveness of these treatments. Since 1990, more people have died from coronary heart disease than from any other cause; ischaemic heart disease claims more lives than all forms of cancer combined [1]. In 2008, 30% of all global deaths (17.3 million) were attributed to ischaemic heart disease, a number that is expected to rise to more than 23.6 million by 2030 [1]. In 2015, projected costs of cardiovascular diseases totalled $656 billions in the US including health expenditures and lost productivity; these costs are expected to double by 2030 (projected total costs $1.208 billions)[2].

At present, more than 4,700 clinical trials investigating cardiovascular disease are listed on the”International Clinical Trials Registry Platform”(ICTRP) of the World Health Organisation [3] involving thousands of patients and costing millions of euro. The results of clinical trials assessing similar interventions are summarised in systematic reviews and meta-analyses providing the basis for guidelines and treatment recommendations. However, it has been reported that clinical endpoints in cardiac interventional research are measured and reported inconsistently [4], limiting the ability of research synthesis [5]. As a consequence, a significant number of studies are regularly excluded from meta-analyses reducing power and limiting the value of evidence [6]. Furthermore, empirical research strongly determines that outcome-reporting bias has significant impact on how the results of clinical trials are reported [7], influencing strongly the implications identified by systematic reviewers.

This problem is minimised by introducing minimum core outcome sets (COS) for a specific clinical field [5,8]. A minimum core outcome set is defined as an agreed minimum set of outcomes that should be measured and reported in all clinical trials for a specific clinical area [5]. COS intend to increase the reporting of outcomes important to all stakeholders, limit study heterogeneity and avoid selective reporting [8]. The use of COS is strongly recommended by international funding bodies [9], and journal editors (CROWN Initiative, http://www.crown-initiative.org). And equally important, the call for COS are in line with the *Clinical Outcome Assessment Qualification Program* by the US Food and Drug Administration (FDA), which promotes the development and implementation of patient-focused endpoint measures in medical product development to describe clinical benefit in labelling [10].

The Core Outcome Measures in Effectiveness Trials (COMET) Initiative and the Outcome Measures in Rheumatology (OMERACT) Initiative [11] suggests a stepwise approach to COS development. OMERACT introduced a framework, which aims to include all key aspects of a health condition (death, life impact, resource use, and pathophysiological manifestations) to ensure the comprehensiveness and applicability of core outcome sets [11]. By doing so, the outcomes and measurement instruments are consistent with the reporting framework currently introduced for the US Clinical Trials Registry. A recently published systematic review [12] on available core outcome sets for comparative effectiveness research highlighted that no COS exists for trials investigating pre-, intra- or postsurgical interventions in conventional cardiac surgery. In light of above, we developed a COS for adult cardiac surgery effectiveness trials.

## Material and methods

The conduct and reporting of this COS adheres, in as much as practicable, to the recommendations given by COMET [8], the methodological guidance provided by Sinha and colleagues [13] on Delphi studies and by the OMERACT Initiative [11]. Our study complies with the Declaration of Helsinki, ethical approval was obtained (Faculty of Medicine, RWTH Aachen, file reference EK 338/14) and a detailed study protocol was composed and published elsewhere [14] (provided as supporting material, S1 File). The reporting of this COS study followed the recommendations of the COS-STAR statement [15], provided as supporting material S2 File.

### Scope of the COS

This COS is intended for clinical trials measuring the effectiveness of pre-, intra- or postsurgical interventions in invasive off- or on-pump cardiac surgery (elective and emergency procedures, excluding transplants, participants > 18 years).

### Preliminary work: Identification of existing knowledge

In preparation of this study, we performed a systematic review to evaluate current clinical research on invasive off- or on-pump cardiac surgery (elective and emergency surgeries, excluding transplants investigating pre-, intra- or postsurgical interventions) to determine the type and number of outcomes reported [4]. Fifteen systematic reviews involving 371 randomised trials and 58,253 patients were analysed. We established unique lists of patient-centred and non salutogenically focused outcomes, which collapsed into 38 outcome categories providing a list of potential core outcomes for the proposed minimum COS.

### Method to reach consensus on the COS (“what” to measure)

We conducted a 3-round eDelphi survey. This facilitated international participation without the time lag between successive rounds associated with traditional postal surveys, enabled a relatively low cost structure, increased data collection efficiencies and offered potential for a higher response rate through rapid communication with participants. The survey was conducted using the online survey software QuestionPro (http://www.questionpro.com).

### Stakeholder involvement and sample size

Participation was sought from people within the following broad groups: adult patients in need or after cardiac surgery, cardio-thoracic surgeons, anaesthesiologists, nursing staff involved with adult cardiac patients, and researchers with expertise in this particular field of research. There is currently no standard method for sample size calculation in Delphi processes, thus a broad approach is taken to facilitate adequate and international participation.

An e-mail inviting participation was sent to the following groups: the German Heart Foundation, the British Cardiac Patients Association, the European Heart Network, the Support Network of the American Heart Association, the German Society for Thoracic and Cardiovascular Surgery (DGTHG), the European Association for Cardio Thoracic Surgery (EACTS), the American Association for Thoracic Surgery (AATS), the Society of Thoracic Surgeons (STS), the Cardiothoracic Surgery Network (CTSNet), the German Society of Anaesthesiology and Intensive Care Medicine, the European Society of Anaesthesiology, the American Society of Anaesthesiologists, and to the Cochrane Heart Group. Participants were invited to forward the invitation to anyone whom they regarded as having the required expertise to substantially contribute to this eDelphi survey. Those who chose to participate were asked to respond with their e-mail address, country of origin and area of expertise. Participants were given information about the study and about core outcome sets. Participants were encouraged to complete the eDelphi questionnaire in each round. An e-mail reminder was send to anyone who did not respond after seven and 14 days.

### Data analyses

#### eDelphi

The first round of the eDelphi contained all outcomes identified by the aforesaid systematic review [4] presented in a randomised manner. Based on the example given by Chiarotto et al. [16], participants were asked to indicate if an outcome was important enough to be included as a core outcome. For all rounds, response options were a) “yes”, b) “no” and c) “unsure / I do not know”. In round 1, participants were encouraged to suggest modifications of definitions/ wording of the outcomes and to indicate if they considered that there was a large conceptual overlap between some outcomes and to judge whether such outcomes could or should be combined. Participants were also asked to identify up to two ‘‘new” outcomes, which they judged to be relevant or important but had not been identified in the systematic review.

Responses to each round were analysed using descriptive statistics. Frequencies on the importance of outcomes were calculated for the whole panel. In addition, we analysed responses from patient representatives separately to assess if they differed from the overall panel rating. We established *a priori* that outcomes for which at least 60% of the participants chose the response option “no” (outcome not important enough to be included as a core outcome) and less than 20% chose the response option “yes” (outcome important enough to be included as a core outcome) would be dropped from the list of potential core outcomes. After each round a report containing the rating for each outcome was sent to each participant.

Based on participants comments, the study team proposed additional aggregation of potential core outcomes after round 1 (“Measure of pulmonary function” and “Measure of pulmonary complications / dysfunction”) as they were similar enough to be combined, and to drop two other outcomes (“Incidence of coronary risk factor” and “Duration of follow up”) from the list of potential core outcomes as they were process variables rather than outcomes. After each round, participants were asked to re-rate each outcome with knowledge of their individual and the group’s previous ratings and to indicate whether or not they agreed with the proposed decisions. The study team set an a priori consensus for inclusion of an outcome in the core outcome set at 67% (“two-thirds majority”). Participants were asked to rate the newly identified outcomes using the same response options. We intended that the identified core outcomes should at best cover all four core areas (death, life impact, resource use and pathophysiological manifestations) of the OMERACT filter 2.0 [11]. Therefore, in round 3 participants were asked to rate which outcome should represent each of the four recommended core areas. The end result of the three-round eDelphi exercise was the proposed COS.

## Results

### Panellists

We invited 1,358 participants to take part in the Delphi study. A total of 86 participants completed the first round of the eDelphi, representing 23 different countries and representing adult patients in need or after cardiac surgery, cardiac surgeons, anaesthesiologists, nursing staff and researchers with expertise in this particular field of research (Table 1).

**Table 1:** Characteristics of participants.

Characteristics	Round 1	Round 2	Round 3
Total number of participants	**86**	**46**	**39**
eDelphi invited	1,358	86	46
viewed	400	109	71
started	147	54	47
completion rate (%)	59%	85%	82%
Stakeholder group n(%), multiple answers were possible			
Adult patient in need or after cardiac surgery	16%	16%	16%
Cardiac surgeon	23%	19%	16%
Anaesthesiologist	10%	15%	15%
Nursing staff involved with adult cardiac patients	5%	2%	2%
Researcher with expertise in this field of research	28%	24%	24%
Other (e.g. cardiologist, physician, perfusionist)	18%	24%	27%
Country of origin (%)			
Argentina	1%	2%	2%
Australia	4%	4%	5%
Belgium	2%	2%	2%
Brazil	1%	2%	2%
Canada	3%	4%	5%
Chile	1%	-	-
China	1%	-	-
Denmark	1%	-	-
France	1%	-	-
Germany	43%	40%	39%
Italy	2%	2%	2%
Macedonia	2%	-	-
Mexico	1%	-	-
Netherlands	1%	-	-
Romania	1%	-	-
Russian Federation	1%	-	-
Saudi Arabia	3%	2%	-
Spain	4%	4%	5%
Switzerland	1%	2%	2%
Syria	1%	2%	2%
United Kingdom	18%	26%	26%
USA	7%	6%	6%
Uruguay	1%	2%	2%

### eDelphi

**Fig 1:**
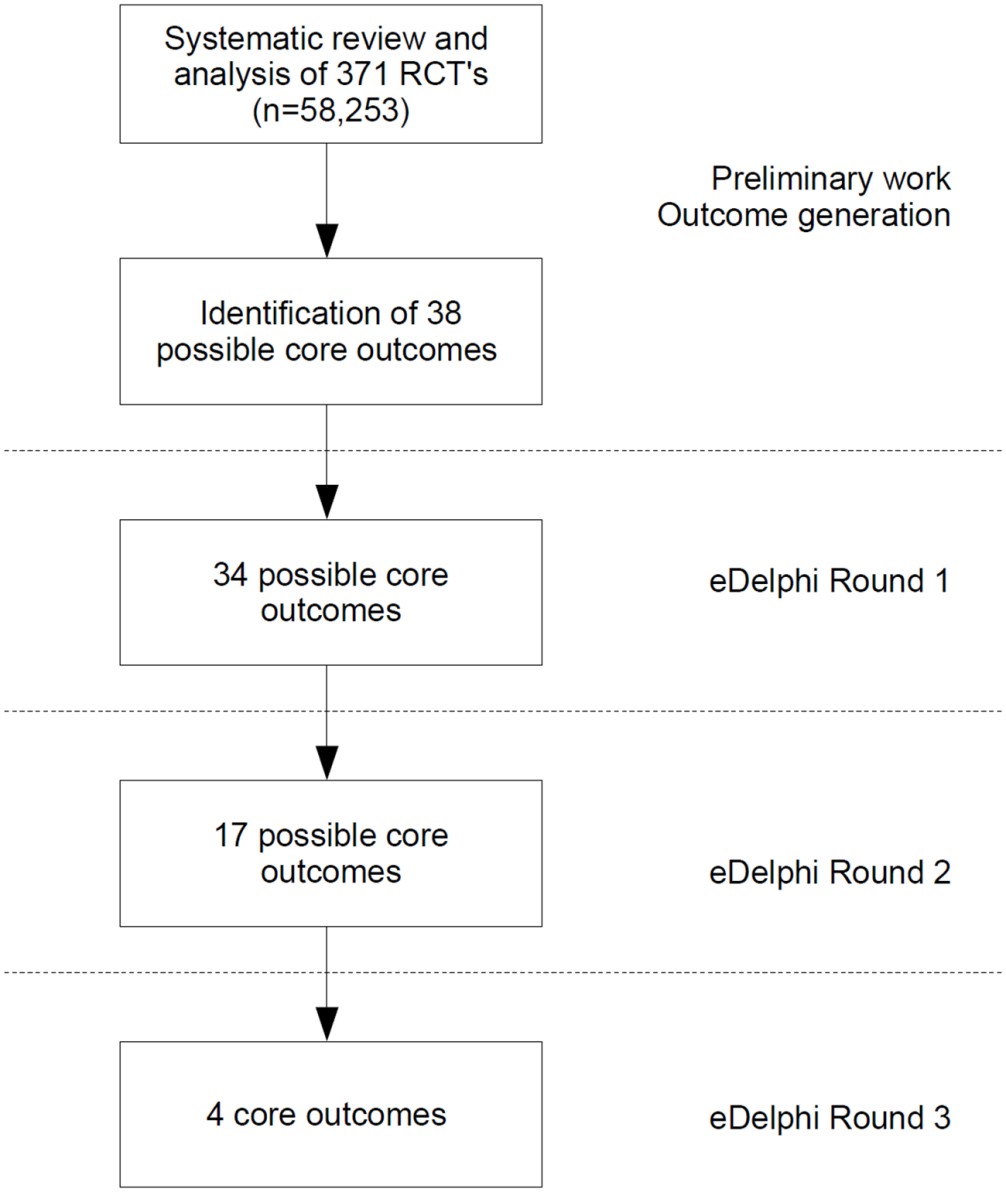
Flow diagram of eDelphi process and core outcome generation.

The subsequent flow diagram (Fig 1) displays the eDelphi process and shows how consent was reached on core outcomes.

#### eDelphi round 1

**Fig 2:**
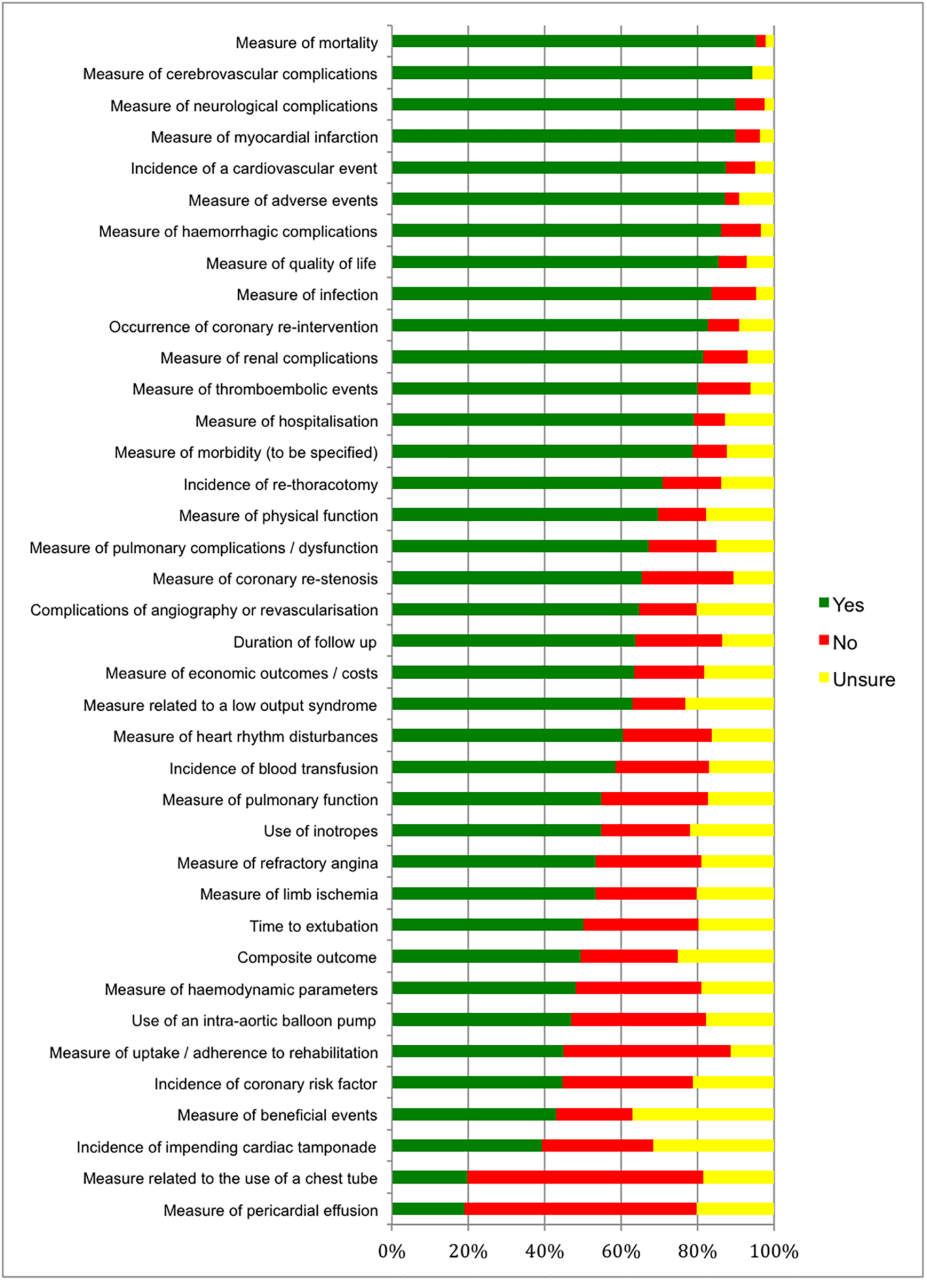
Round 1 of eDelphi: Rating for 38 potential core outcomes.

Fig 2 displays the results of round 1 using bar graphs. We attach as supporting information S1 Table, which shows the rating for all 38 outcomes identified in the systematic review. Two outcomes (“Measure of pericardial effusion” and “Measure related to the use of a chest tube”) were dropped after round 1 in accordance with our pre-specified criteria (at least 60% of the participants chose the response option “no” and less than 20% chose the response option “yes”). In addition, the panel suggested to drop two other outcomes (“Incidence of coronary risk factor” and “Duration of follow up”) from the list of potential core outcomes as they were process variables rather than outcomes and to combine two outcomes (“Measure of pulmonary function” and “Measure of pulmonary complications / dysfunction”) into one outcome as they were similar enough to be combined, which was confirmed in round 2 and implemented in the list in round 3 of the eDelphi.

#### eDelphi round 2

**Fig 3:**
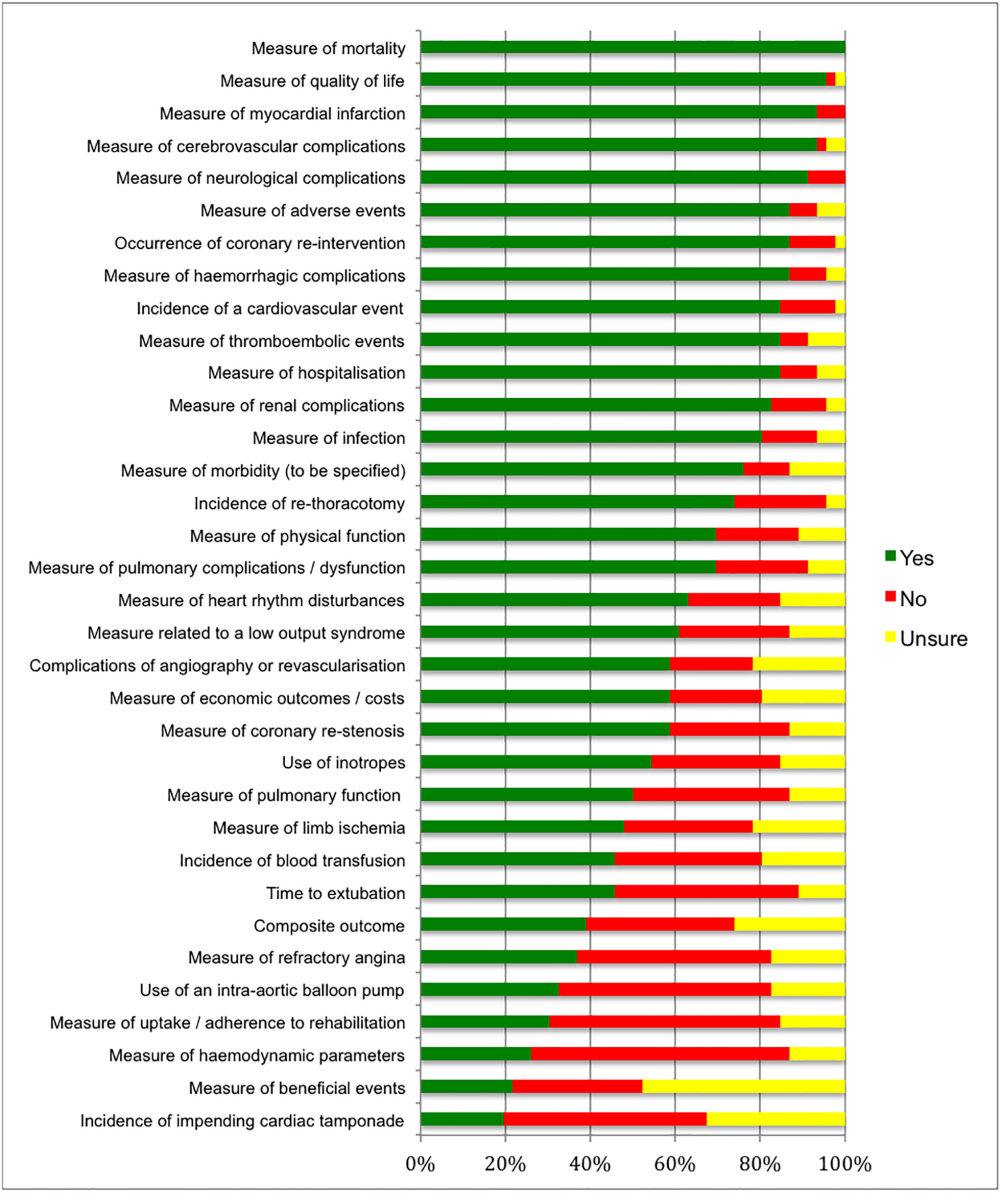
Round 2 of eDelphi: Rating for 34 potential core outcomes.

Supporting information S2 Table shows the rating for all 34 outcomes included in round 2 of the eDelphi; Fig 3 displays the results using bar graphs. Following analysis of round 2 responses, 17 outcomes were removed leaving 17 outcomes in the round 3 survey.

#### eDelphi round 3

Supporting information S3–S6 Tables and S1–S4 Figs show the rating for the 17 remaining outcomes from round 3 of the eDelphi. As we intended that the identified core outcomes would cover the recommended core areas (death, life impact, resource use and pathophysiological manifestations), the panel rated which of the 17 remaining core outcomes would at best represent each of the four recommended core areas. For all four core areas, agreement was high on which outcome should represent the core area. Table 2 presents the final agreed “COS Adult Cardiac Surgery” for cardiac surgery effectiveness trials.

**Table 2:** Final “COS Adult Cardiac Surgery”.

Core outcomes
Mortality
Quality of life
Hospitalisation
Cerebrovascular complication

## Discussion

This study used robust methods to develop the first COS relevant to cardiac surgery effectiveness trials. We recommend that corresponding trials include the outcomes mortality, quality of life, hospitalisation and cerebrovascular complications in the list of outcomes they assess during the course of their trial. This “COS Adult Cardiac Surgery” will limit reporting bias and heterogeneity across trials in cardiac surgery. The involvement of multiple stakeholders and the application of agreed methodological aspects of COS development assures the applicability and dissemination of the developed COS.

As a next step, measurement instruments need to be identified to measure the core outcomes of the “COS Adult Cardiac Surgery”. For applicability, each instrument must prove to be valid, discriminative and feasible. We will systematically search and appraise possible matching measurement instruments for each of the agreed core outcomes. Where only partially validated instruments are identified, or where no instruments are available, instruments will need to be further validated, or developed, and their applicability documented. Where no instruments are available, these will need to be developed. To describe next steps in more detail, we would like to highlight these on the basis of the core outcome “Quality of life”. We are currently undertaking a study that compares available quality of life measurement tools in the described population. We performed a systematic search to identify available measurement tools for the cardiac surgical population (e.g. MacNew Heart Disease health-related quality of life instrument) and included in our study also most commonly used measurement tools for quality of life (SF-36) to allow direct comparison between available tools. The results of this study will then form the basis for expert consent on the outcome measurement tool most applicable to measure quality of life in the considered population.

### Implementation and updating of this COS

We registered this COS with the COMET database (http://www.comet-initiative.org/studies/details/630?result=true). To increase the uptake of this COS, we engaged with the Cochrane Heart Group and with clinical guideline developers such as the American Heart Association and the German Society for Thoracic and Cardiovascular Surgery. This “COS Adult Cardiac Surgery” will be introduced at national and international conferences. Reviewing a COS regularly is important as a form of validation, to ensure outcomes are still important to all stakeholders, to give the chance to add new outcomes, to evaluate how successful implementation has been and to engage further stakeholders if suitable [8]. The question of who should review a COS in what timeframes needs careful consideration. So far, no recommendations exist. Our study group will revisit this issue regularly and follow up on this topic within two years.

We want to acknowledge one slight difference between our study and the study protocol [14]. Various groups have used the terms “core outcome set” and “outcome measure” differently, disagreement is continuing and no uniform definition is universally acknowledged. The COMET Initiative now defines a COS as “an agreed minimum set of outcomes or outcome measures”(http://www.comet-initiative.org), while previously a COS was defined as a “an agreed minimum set of outcomes that should be measured and reported identically in clinical trials for a specific clinical area” [5]. Furthermore, COMET specifies that a COS is a recommendation of “*what*” to measure and that researchers also need to consider “*how*” these outcomes should be measured and refer to the “*how*” as a “Core Outcome Measurement Instrument Set”. However, the OMERACT Initiative uses the term “outcome domain” to describe the “*what*” and the term “Core Outcome Set” to describe the “*how*”. Within our study protocol we followed the definitions used by OMERACT, but as the definitions by COMET become more widely used and are more acknowledged [17], we adopted them within this manuscript.

There are some limitations to our study. As there is currently no standard method for sample size calculations for Delphi studies, we took a broad approach (minimising selection bias) to ensure international participation in this Delphi study, but of the 1,358 participants invited to take part in this study only 86 (6.33%) did so. The response rates of cardiac patients were especially low, but nevertheless nearly 20% of the overall panel consisted of patients. It has been argued that patients’ views should be given greater value in COS development [8,11]. Therefore, we analysed responses from patient representatives separately to assess if they differed from the general panel rating. By doing so we acknowledged the importance of patients compared to those of health professionals. However, we did not observe a significant difference in judgement between patients and other stakeholders and we believe that all of our recommendations are justified on the basis of the evidence we have cited.

It is also important to note that when analysing data of 198 already available COS studies, Gargon and colleagues highlighted, that of 198 identified COS studies only 20 studies included patient representatives in the panel. Of those, more than half had less patient representatives than our COS study.

## Supporting information

S1 File Study protocol. (PDF)

S2 File COS-STAR checklist. (DOC)

S1 Table Results of eDelphi round 1. (DOCX)

S2 Table Results of eDelphi round 2. (DOCX)

S3 Table Results of eDelphi round 3, core area death. (DOCX)

S4 Table Results of eDelphi round 3, core area life impact. (DOCX)

S5 Table Results of eDelphi round 3; core area resource use/economical impact. (DOCX)

S6 Table Results of eDelphi Round 3; core area pathophysiological manifestation. (DOCX)

S1 Fig Round 3 of eDelphi: Rating for 17 potential core outcomes, core area death. (TIF)

S2 Fig Round 3 of eDelphi: Rating for 17 potential core outcomes, core area life impact. (TIF)

S3 Fig Round 3 of eDelphi: Rating for 17 potential core outcomes, core area resource use/economical impact. (TIF)

S4 Fig Round 3 of eDelphi: Rating for 17 potential core outcomes, core area pathophysiological manifestation. (TIF)
