# Supplementary material for: A core outcome set for adult cardiac surgery trials: A consensus study
Source: PLoS One. 2017 Nov 2;12(11):e0186772. doi: 10.1371/journal.pone.0186772 (PMC5667757; doi:10.1371/journal.pone.0186772)
Supplement: S2 File — (DOC) [file pone.0186772.s002.doc]

**COS-STAR Statement, Checklist**

“**A Core Outcome Set for Adult Cardiac Surgery Trials: a Consensus Study”**

| **SECTION/TOPIC** | **ITEM**  **No.** | **CHECKLIST ITEM** | **To find within the manuscript** |
| --- | --- | --- | --- |
| **TITLE/ABSTRACT** |  |  |  |
| Title | 1a | Identify in the title that the paper reports the development of a COS | ✓ |
| Abstract | 1b | Provide a structured summary | ✓  pp. 2-3 |
| **INTRODUCTION** |  |  |  |
| Background and  Objectives | 2a | Describe the background and explain the rationale for developing the  COS. | ✓  pp. 4-5 |
|  | 2b | Describe the specific objectives with reference to developing a COS. | ✓  p. 6 |
| Scope | 3a | Describe the health condition(s) and population(s) covered by the COS. | ✓  pp. 6 |
|  | 3b | Describe the intervention(s) covered by the COS. | ✓  p. 6 |
|  | 3c | Describe the setting(s) in which the COS is to be applied. | ✓  p. 6 |
| **METHODS** |  |  |  |
| Protocol/Registry Entry | 4 | Indicate where the COS development protocol can be accessed, if available, and/or the study registration details. | ✓  p. 5 |
| Participants | 5 | Describe the rationale for stakeholder groups involved in the COS development process, eligibility criteria for participants from each group, and a description of how the individuals involved were identified. | ✓  p.7 |
| Information Sources | 6a | Describe the information sources used to identify an initial list of outcomes. | ✓  pp. 6 |
|  | 6b | Describe how outcomes were dropped/combined, with reasons (if applicable). | ✓  pp. 11-15 |
| Consensus Process | 7 | Describe how the consensus process was undertaken. | ✓  pp. 7-9 |
| Outcome Scoring | 8 | Describe how outcomes were scored and how scores were summarised. | ✓  pp. 7-9 |
| Consensus Definition | 9a | Describe the consensus definition. | ✓  pp. 8-9 |
|  | 9b | Describe the procedure for determining how outcomes were included or excluded from consideration during the consensus process. | ✓  pp. 7-9 |
| Ethics and Consent | 10 | Provide a statement regarding the ethics and consent issues for the study. | ✓  p. 5 |
| **RESULTS** |  |  |  |
| Protocol Deviations | 11 | Describe any changes from the protocol (if applicable), with reasons, and describe what impact these changes have on the results. | ✓  pp. 16-17 |
| Participants | 12 | Present data on the number and relevant characteristics of the people involved at all stages of COS development. | ✓  pp. 9-11 |
| Outcomes | 13a | List all outcomes considered at the start of the consensus process. | ✓  p. 13 |
|  | 13b | Describe any new outcomes introduced and any outcomes dropped, with reasons, during the consensus process. | ✓  pp. 12-14 |
| **COS** | 14 | List the outcomes in the final COS. | ✓  p. 15 |
| **DISCUSSION** |  |  |  |
| Limitations | 15 | Discuss any limitations in the COS development process. | ✓  p. 17 |
| Conclusions | 16 | Provide an interpretation of the final COS in the context of other evidence, and implications for future research. | ✓  pp. 16-18 |
| **OTHER**  **INFORMATION** |  |  |  |
| Funding | 17 | Describe sources of funding/role of funders. | ✓  p. 18 |
| Conflicts of Interest | 18 | Describe any conflicts of interest within the study team and how these were managed. | ✓  p. 18 |
