# Supplementary material for: A core outcome set for adult cardiac surgery trials: A consensus study
Source: PLoS One. 2017 Nov 2;12(11):e0186772. doi: 10.1371/journal.pone.0186772 (PMC5667757; doi:10.1371/journal.pone.0186772)
Supplement: S1 Table — (DOCX) [file pone.0186772.s003.docx]

**S1 Table. Results of eDelphi Round 1**

| **Potential core outcomes** | **Yes (%)** | **No (%)** | **Unsure (%)** |
| --- | --- | --- | --- |
| Time to extubation | 43(50.00) | 26(30.23) | 17(19.77) |
| Measure of cerebrovascular complications | 81(94.19) | 0(0.00) | 5(5.81) |
| Measure of beneficial events | 37(43.02) | 17(19.779) | 32(37.21) |
| Use of inotropes | 47(54.65) | 20(23.26) | 19(22.09) |
| Measure of pericardial effusion* | 16(19.05) | 51(60.71) | 17(20.24) |
| Measure of renal complications | 70(81.40) | 10(11.63) | 6(6.98) |
| Measure of haemorrhagic complications | 74(86.05) | 9(10.47) | 3(3.49) |
| Measure of hospitalisation | 68(79.07) | 7(8.14) | 11(12.79) |
| Measure related to a low output syndrome | 54(62.79) | 12(13.95) | 20(23.26) |
| Measure of pulmonary function | 47(54.65) | 24(27.91) | 15(17.44) |
| Measure related to the use of a chest tube* | 17(19.77) | 53(61.63) | 16(18.60) |
| Measure of mortality | 82(95.35) | 2(2.33) | 2(2.33) |
| Measure of heart rhythm disturbances | 52(60.47) | 20(23.26) | 14(16.28) |
| Occurrence of coronary re-intervention | 71(82.56) | 7(8.14) | 8(9.30) |
| Measure of adverse events | 75(87.21) | 3(3.49) | 8(9.30) |
| Incidence of coronary risk factor* | 38(44.71) | 29(34.12) | 18(21.18) |
| Measure of infection | 71(83.53) | 10(11.76) | 4(4.71) |
| Measure of coronary re-stenosis | 55(65.48) | 20(23.81) | 9(10.71) |
| Measure of economic outcomes / costs | 52(63.41) | 15(18.29) | 15(18.29) |
| Measure of quality of life | 70(85.37) | 6(7.32) | 6(7.32) |
| Incidence of blood transfusion | 48(58.54) | 20(24.39) | 14(17.07) |
| Duration of follow up* | 51(63.75) | 18(22.50) | 11(13.75) |
| Measure of thromboembolic events | 64(80.00) | 11(13.75) | 5(6.25) |
| Measure of morbidity (to be specified) | 63(78.75) | 7(8.75) | 10(12.50) |
| Measure of limb ischemia | 42(53.16) | 21(26.58) | 16(20.25) |
| Complications of angiography or revascularisation | 51(64.56) | 12(15.19) | 16(20.25) |
| Composite outcome | 39(49.37) | 20(25.32) | 20(25.32) |
| Incidence of re-thoracotomy | 56(70.89) | 12(15.19) | 11(13.92) |
| Measure of pulmonary complications / dysfunction | 53(67.09) | 14(17.72) | 12(15.19) |
| Incidence of a cardiovascular event | 69(87.34) | 6(7.59) | 4(5.06) |
| Measure of haemodynamic parameters | 38(48.10) | 26(32.91) | 15(18.99) |
| Measure of refractory angina | 42(53.16) | 22(27.85) | 15(18.99) |
| Measure of uptake / adherence to rehabilitation and lifestyle | 35(44.87) | 34(43.59) | 9(11.54) |
| Measure of physical function | 55(69.62) | 10(12.66) | 14(17.72) |
| Incidence of impending cardiac tamponade | 31(39.24) | 23(29.11) | 25(31.65) |
| Use of an intra-aortic balloon pump | 37(46.84) | 28(35.44) | 14(17.72) |
| Measure of myocardial infarction | 71(89.87) | 5(6.33) | 3(3.80) |
| Measure of neurological complications | 71(89.87) | 6(7.59) | 2(2.53) |

***** Two outcomes (“Measure of pericardial effusion” and “Measure related to the use of a chest tube”) were dropped after round 1 in accordance with our pre-specified criteria (at least 60% of the participants chose the response option “no” and less than 20% chose the response option “yes”). In addition, the panel suggested to drop two other outcomes (“Incidence of coronary risk factor” and “Duration of follow up”) from the list of potential core outcomes as they were process variables rather than outcomes and to combine two outcomes (“Measure of pulmonary function” and “Measure of pulmonary complications / dysfunction”) into one outcome as they were similar enough to be combined.
