# Supplementary material for: A core outcome set for adult cardiac surgery trials: A consensus study
Source: PLoS One. 2017 Nov 2;12(11):e0186772. doi: 10.1371/journal.pone.0186772 (PMC5667757; doi:10.1371/journal.pone.0186772)
Supplement: S2 Table — (DOCX) [file pone.0186772.s004.docx]

**S2 Table. Results of eDelphi Round 2**

| **Potential core outcomes** | **Yes (%)** | **No (%)** | **Unsure (%)** |
| --- | --- | --- | --- |
| Time to extubation* | 21(45.65) | 20(43.48) | 5(10.87) |
| Measure of cerebrovascular complications | 43(93.48) | 1(2.17) | 2(4.35) |
| Measure of beneficial events* | 10(21.74) | 14(30.43) | 22(47.83) |
| Use of inotropes* | 25(54.35) | 14(30.43) | 7(15.22) |
| Measure of renal complications | 38(82.61) | 6(13.04) | 2(4.35) |
| Measure of haemorrhagic complications | 40(86.96) | 4(8.70) | 2(4.35) |
| Measure of hospitalisation | 39(84.78) | 4(8.70) | 3(6.52) |
| Measure related to a low output syndrome* | 28(60.87) | 12(26.09) | 6(13.04) |
| Measure of pulmonary function * | 23(50.00) | 17(36.96) | 6(13.04) |
| Measure of mortality | 46(100.00) | 0(0.00) | 0(0.00) |
| Measure of heart rhythm disturbances* | 29(63.04) | 10(21.74) | 7(15.22) |
| Occurrence of coronary re-intervention | 40(86.96) | 5(10.87) | 1(2.17) |
| Measure of adverse events | 40(86.96) | 3(6.52) | 3(6.52) |
| Measure of infection | 37(80.43) | 6(13.04) | 3(6.52) |
| Measure of coronary re-stenosis* | 27(58.70) | 13(28.26) | 6(13.04) |
| Measure of economic outcomes / costs* | 27(58.70) | 10(21.74) | 9(19.57) |
| Measure of quality of life | 44(95.65) | 1(2.17) | 1(2.17) |
| Incidence of blood transfusion* | 21(45.65) | 16(34.78) | 9(19.57) |
| Measure of thromboembolic events | 39(84.78) | 3(6.52) | 4(8.70) |
| Measure of morbidity (to be specified) | 35(76.09) | 5(10.87) | 6(13.04) |
| Measure of limb ischemia* | 22(47.83) | 14(30.43) | 10(21.74) |
| Complications of angiography or revascularisation* | 27(58.70) | 9(19.57) | 10(21.74) |
| Composite outcome* | 18(39.13) | 16(34.78) | 12(26.09) |
| Incidence of re-thoracotomy | 34(73.91) | 10(21.74) | 2(4.35) |
| Measure of pulmonary function / complications / dysfunction | 32(69.57) | 10(21.74) | 4(8.70) |
| Incidence of a cardiovascular event | 39(84.78) | 6(13.04) | 1(2.17) |
| Measure of haemodynamic parameters* | 12(26.09) | 28(60.87) | 6(13.04) |
| Measure of refractory angina* | 17(36.96) | 21(45.65) | 8(17.39) |
| Measure of uptake / adherence to rehabilitation and lifestyle* | 14(30.43) | 25(54.35) | 7(15.22) |
| Measure of physical function | 32(69.57) | 9(19.57) | 5(10.87) |
| Incidence of impending cardiac tamponade* | 9(19.57) | 22(47.83) | 15(32.61) |
| Use of an intra-aortic balloon pump* | 15(32.61) | 23(50.00) | 8(17.39) |
| Measure of myocardial infarction | 43(93.48) | 3(6.52) | 0(0.00) |
| Measure of neurological complications | 42(91.30) | 4(8.70) | 0(0.00) |

*Outcomes excluded after round 2
