# Supplementary material for: A core outcome set for adult cardiac surgery trials: A consensus study
Source: PLoS One. 2017 Nov 2;12(11):e0186772. doi: 10.1371/journal.pone.0186772 (PMC5667757; doi:10.1371/journal.pone.0186772)
Supplement: S5 Table — (DOCX) [file pone.0186772.s007.docx]

**S5 Table. Results of eDelphi Round 3; core area RESOURCE USE/ECONOMICAL IMPACT**

| **Potential core outcomes** | **Yes (%)** | **No (%)** | **Unsure (%)** |
| --- | --- | --- | --- |
| Measure of cerebrovascular complications | 25(64.10) | 14(35.90) | 0(0.00) |
| Measure of renal complications | 24(61.54) | 15(38.46) | 0(0.00) |
| Measure of haemorrhagic complications | 21(53.85) | 17(43.59) | 1(2.56) |
| Measure of hospitalisation* | 37(94.87) | 1(2.56) | 1(2.56) |
| Measure of mortality | 26(66.67) | 12(30.77) | 1(2.56) |
| Occurrence of coronary re-intervention | 32(82.05) | 7(17.95) | 0(0.00) |
| Measure of adverse events | 27(69.23) | 10(25.64) | 2(5.13) |
| Measure of infection | 26(66.67) | 12(30.77) | 1(2.56) |
| Measure of quality of life | 20(51.28) | 18(46.15) | 1(2.56) |
| Measure of thromboembolic events | 23(58.97) | 14(35.90) | 2(5.13) |
| Measure of morbidity (to be specified) | 24(61.54) | 12(30.77) | 3(7.69) |
| Incidence of re-thoracotomy | 26(66.67) | 13(33.33) | 0(0.00) |
| Measure of pulmonary function/ complications/ dysfunction | 18(46.15) | 21(53.85) | 0(0.00) |
| Incidence of a cardiovascular event | 23(58.97) | 16(41.03) | 0(0.00) |
| Measure of physical function | 20(51.28) | 18(46.15) | 1(2.56) |
| Measure of myocardial infarction | 24(61.54) | 15(38.46) | 0(0.00) |
| Measure of neurological complications | 26(66.67) | 13(33.33) | 0(0.00) |

*Core Outcome
