# Supplementary figures and images for: A core outcome set for adult cardiac surgery trials: A consensus study
Source: PLoS One. 2017 Nov 2;12(11):e0186772. doi: 10.1371/journal.pone.0186772 (PMC5667757; doi:10.1371/journal.pone.0186772)

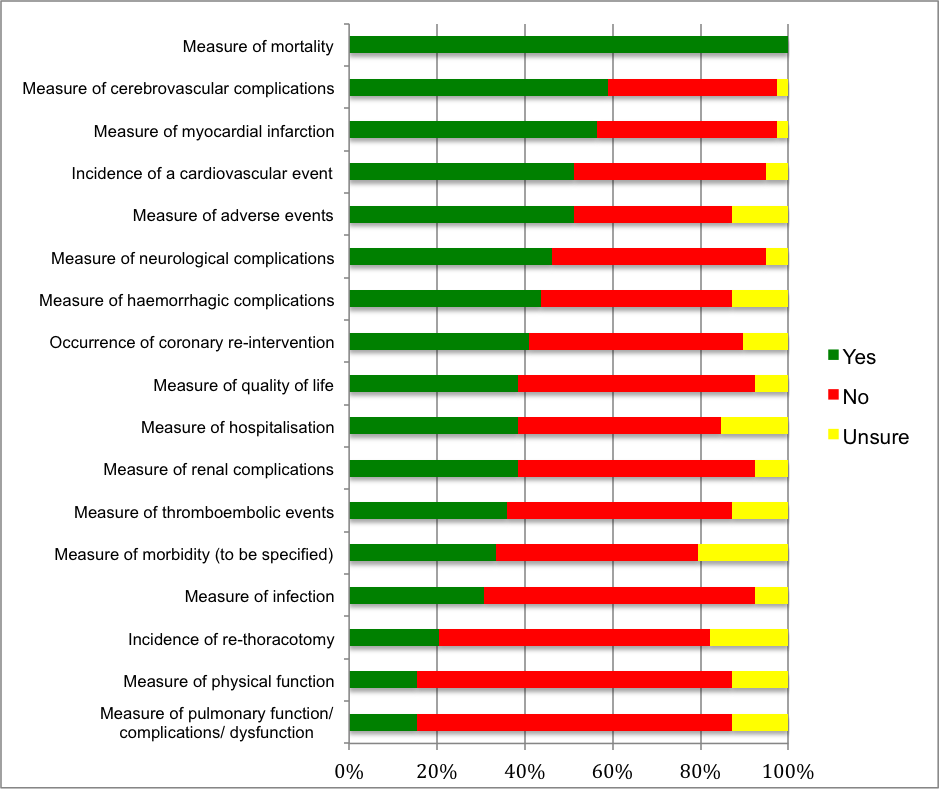

Supplement: S1 Fig — (TIF) [file pone.0186772.s009.tif]

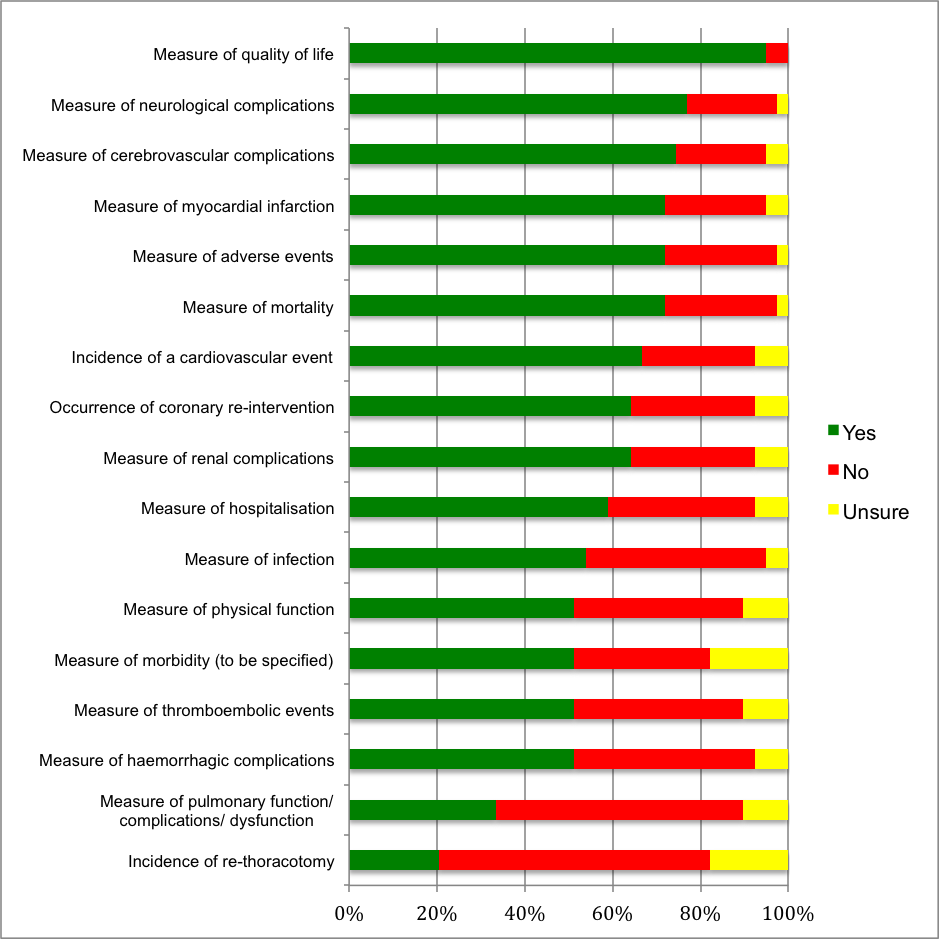

Supplement: S2 Fig — (TIF) [file pone.0186772.s010.tif]

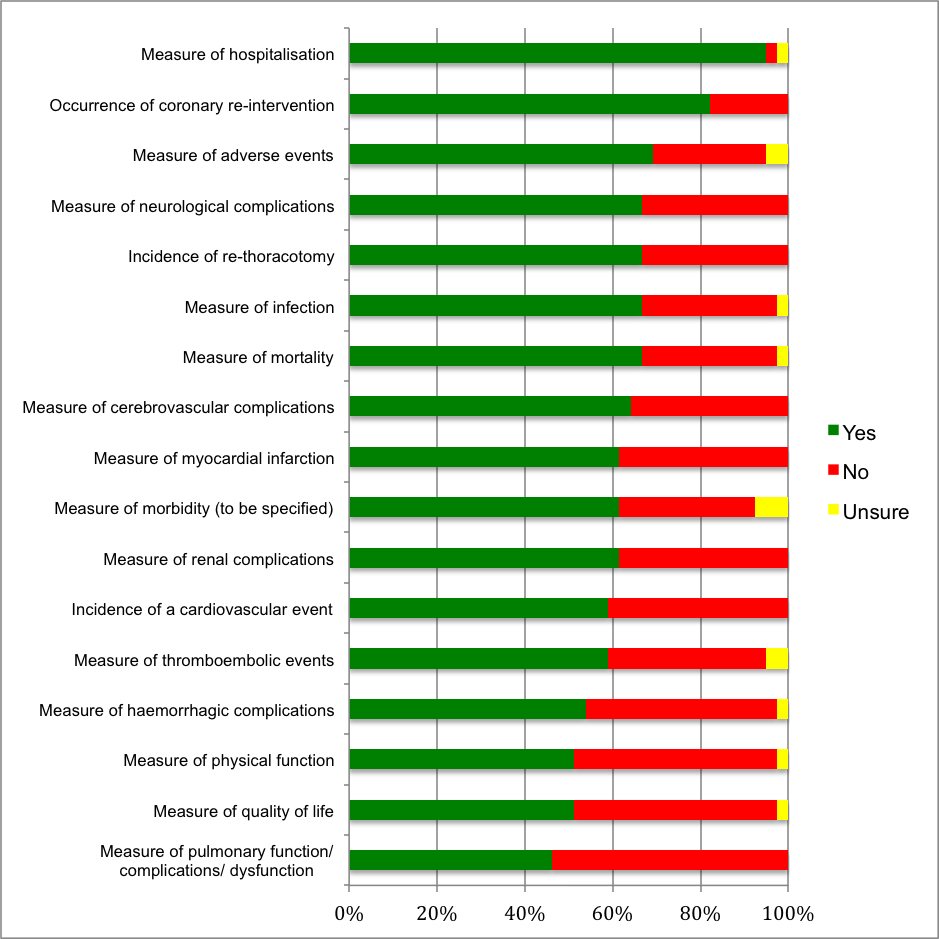

Supplement: S3 Fig — (TIF) [file pone.0186772.s011.tif]

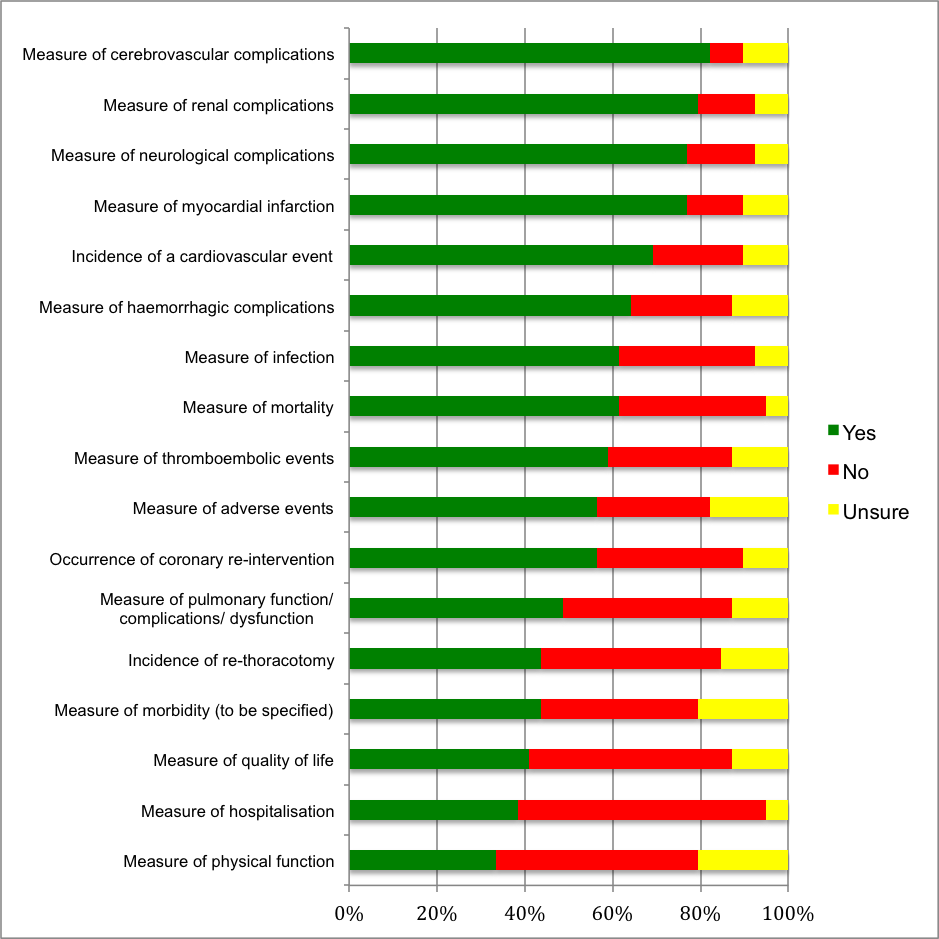

Supplement: S4 Fig — (TIF) [file pone.0186772.s012.tif]
